# Study on the therapeutic effect of Kirschner wire tension band combined with anchor cross-stitch technique in the treatment of comminuted patellar inferopolar fractures

**DOI:** 10.1371/journal.pone.0302839

**Published:** 2024-05-02

**Authors:** SiYu Duan, He Zhang, HaiRui Liang, RongDa Xu, Ming Sun, Hanfei Liu, XueTing Zhou, Hang Wen, ZhenCun Cai

**Affiliations:** 1 Department of Orthopedic Surgery, The Affiliated Central Hospital of Shenyang Medical College, Shenyang City, Liaoning Province, China; 2 Key Laboratory of Human Ethnic Specificity and Phenomics of Critical Illness in Liaoning Province, Shenyang Medical College, Shenyang City, China; Sheikh Hasina National Institute of Burn & Plastic Surgery, BANGLADESH

## Abstract

**Purposes:**

Fractures of the inferior patellar pole, unlike other patellar fractures, present challenges for traditional surgical fixation methods. This article introduces the clinical technique and outcomes of using Kirschner wire tension band combined with anchor screw cross-stitch fixation for comminuted inferior patellar pole fractures.

**Methods:**

This retrospective case series study included 14 patients with comminuted inferior patellar pole fractures treated at our institution from September 1, 2020, to April 30, 2022. All patients underwent surgery using the Kirschner wire tension band with anchor screw cross-stitch technique. Follow-up assessments involved postoperative X-rays to evaluate fracture healing, as well as clinical parameters such as healing time, Visual Analog Scale (VAS) scores, range of motion (ROM), and Bostman scores.

**Results:**

All patients were followed for an average of over 12 months, with no cases of internal fixation failure. Knee joint stability and function were excellent. X-rays revealed an average healing time of approximately 10.79 ± 1.53 weeks, hospitalization lasted 5.64 ± 1.15 days, surgery took approximately 37.86 ± 5.32 minutes, and intraoperative blood loss was 33.29 ± 8.15 ml. One patient experienced irritation from the internal fixation material. At the final follow-up, the Bostman score averaged 28.29 ± 0.83, knee joint flexion reached 131.07° ± 4.88°, all patients achieved full knee extension, and the VAS score was 0.36 ± 0.63.

**Conclusion:**

Kirschner wire tension band with anchor screw cross-stitch fixation for comminuted inferior patellar pole fractures delivered satisfactory clinical outcomes. This surgical method, characterized by its simplicity and reliability, is a valuable addition to clinical practice.

## 1. Introduction

The patella, the largest sesamoid bone in the human body, comprises approximately 1% of total body fractures [1,2]. The inferior pole of the patella serves as the attachment point of the patellar ligament, reinforcing the force arm of the quadriceps femoris muscle. This area experiences concentrated stress and is a crucial component of the knee extension mechanism. Therefore, the integrity of the patella plays a vital role in the functionality of the knee joint. The inferior pole of the patella is primarily composed of cancellous bone without cartilaginous covering on its surface. Fractures in this area are relatively uncommon, accounting for approximately 9.3% to 22.4% of all patellar fractures, and they usually occur due to direct force or are more prevalent in patients with osteoporosis [3,4]. Fractures of the inferior patellar pole lead to impaired knee extension function and typically require surgical treatment. The objective is to restore the integrity and stability of the knee extension mechanism, aiming to achieve anatomical reduction as much as possible, thereby facilitating early and effective rehabilitation exercises to reduce the occurrence of fracture complications [5]. With the development of medical technology, the surgical methods for fractures of the inferior patellar pole have gradually become diverse, including Kirschner wire tension band fixation, cerclage wiring, screw fixation, anchor fixation, suture fixation, and inferopolar patellar resection [4,6–10]. As of now, there is no consensus on the treatment approach for comminuted fractures of the inferior patellar pole in clinical practice [11]. In our clinical practice, we have found that combining anchor screw cross-stitch fixation with Kirschner wire tension band fixation significantly enhances the therapeutic effect in the treatment of comminuted fractures of the inferior patellar pole. This approach provides effective physical stability for fracture healing, and the procedure is simple and easy to perform. Herein, we report on patients with comminuted fractures of the inferior patellar pole treated at our hospital from September 2020 to April 2022. The aim of this study is to analyze the clinical effectiveness of combining Kirschner wire tension band with anchor screw cross-stitch fixation.

## 2. Clinical data and methods

### 2.1. General information

A retrospective analysis was conducted on patients with patellar fractures admitted to the Affiliated Central Hospital of Shenyang Medical College from September 2020 to April 2022. This study has obtained approval from the Ethics Review Committee of the Affiliated Central Hospital of Shenyang Medical College (Ethics Approval Number: 2022018), and written informed consent was obtained from all patients. Inclusion criteria for patients were as follows: (1) normal knee function before injury, closed unilateral patellar fracture; (2) fractures located in the inferior pole of the patella, with comminuted fracture fragments ≥3 pieces; (3) surgery performed within 7 days after injury; (4) no apparent surgical contraindications assessed preoperatively. Exclusion criteria included: (1) concomitant fractures in other parts of the ipsilateral limb; (2) severe osteoporosis and other diseases affecting normal knee joint movement; (3) open fractures; (4) loss to follow-up. According to the inclusion and exclusion criteria, a total of 14 patients were included in this study, including 9 males and 5 females, with an age range of 26 to 60 years. The mechanisms of injury included 10 cases of falls, 3 cases of motor vehicle accidents, and 1 case of falling. The fractures were classified as AO type 34-A1, and the surgical timing ranged from 2 to 7 days after injury, with a minimum follow-up duration of no less than 12 months.

### 2.2. Preoperative preparation

After admission, all patients underwent preoperative X-ray and three-dimensional CT scans to complete the preoperative evaluation. The injured lower limb of the patients was temporarily immobilized in extension at 0° using a plaster cast to alleviate pain. Limb elevation was maintained, and routine pain relief and anticoagulation drugs were administered preoperatively. After excluding surgical contraindications, surgical treatment was performed. All patients received a single dose of antibiotics 30 minutes before surgery. The surgeries were performed by the same experienced group of surgeons, and the surgical anchors used were provided by Smith & Nephew.

### 2.3. Surgical procedure

**Fig 1:**
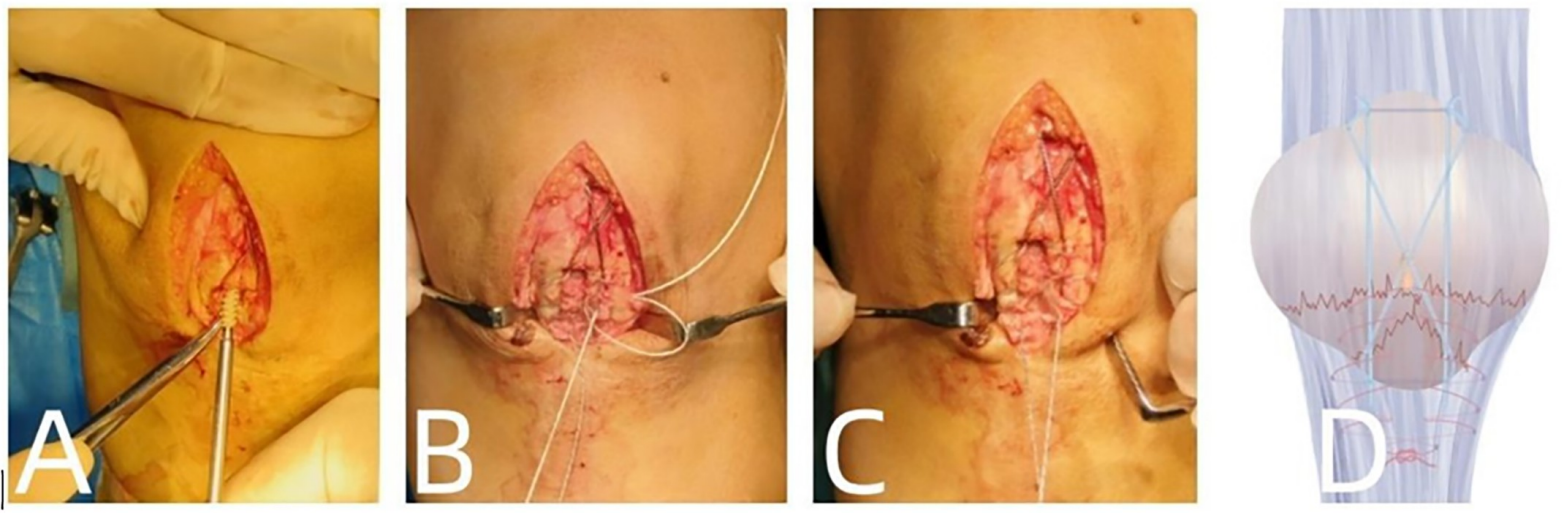
**A:** Insert the cable tie anchor vertically into the transverse section of the proximal patellar fracture. **B, C:** Use suture anchors to cross-stitch and secure the fractured patellar inferopolar fragment to the patellar ligament. **D:** Schematic diagram illustrating the crisscross fixation of the patellar inferopolar fracture using the cable tie anchor.

After the general anesthesia or spinal anesthesia takes effect, the patient is placed in the supine position with a pneumatic tourniquet applied to the proximal thigh. The affected limb is routinely disinfected with iodine. Following hemostasis, assessment for reduction is performed based on preoperative X-ray imaging. A straight anterior incision of approximately 7cm in length is made over the knee joint. The skin, subcutaneous tissue, and fascia are sequentially dissected to expose the shattered bone fragments at the inferior pole of the patella. Saline irrigation is used to cleanse the area, and soft tissues and blood clots at the fracture site are debrided. The joint cavity is irrigated, and the fracture is reduced to restore the integrity of the articular surface. The reduction is then stabilized using a patella reduction clamp or towel clip. During the operation, avoid stripping the patellar tendon and periosteum to prevent separation of fracture fragments. From superior to inferior of the patella, two parallel Kirschner wires (diameter 2.0mm) are inserted, and the steel wire is passed through the ends of the two Kirschner wires, forming a longitudinal figure-eight tension band on the anterior aspect of the patella. Subsequently, the stainless steel wire is tensioned using a Kirschner wire tensioner, and the upper ends of the Kirschner wires are bent over the superior aspect of the patella to prevent loosening and dislodgement. Under C-arm fluoroscopy, following satisfactory reduction of the patellar fracture fragments, a single anchor pin is inserted perpendicular to the transverse plane of the proximal patellar fracture fragment (Fig 1A). The anchor pin is embedded into the body of the patella. The anchor pin tail is then crossed and sutured using a technique, fixing the inferior pole patellar fracture fragment and patellar ligament (Fig 1B–1D). Upon flexion and extension of the knee joint, the fracture remains stable, and the tension of the patellar ligament is intact. Subsequent C-arm fluoroscopy confirms satisfactory alignment of the fracture ends. Physiological saline solution is used to irrigate the surgical site, followed by layer-by-layer suturing of the fascia, subcutaneous tissue, and skin (Fig 1).

### 2.4. Postoperative management

The patient had the plaster external fixation removed postoperatively, received a single dose of prophylactic antibiotics within 24 hours to prevent infection, and underwent routine pain management and anticoagulation therapy. On the first day postoperatively, after reviewing the anteroposterior and lateral X-ray of the knee joint, isometric contraction exercises for the quadriceps femoris, straight leg raising, and progressive knee joint flexion exercises were initiated. The surgical incision is dressed every 2–3 days to observe wound healing, and the stitches are removed 2 weeks after the surgery. Three days post-surgery, the patient is allowed to bear mild weight with the aid of crutches and perform knee flexion exercises. Full range of motion and full weight-bearing without walking aids begin after 4 weeks, progressing to complete knee flexion. For older patients or those with severe comminuted fractures or significant osteoporosis, the duration of crutch use should be extended appropriately.

### 2.5. Assessment criteria

**Fig 2:**
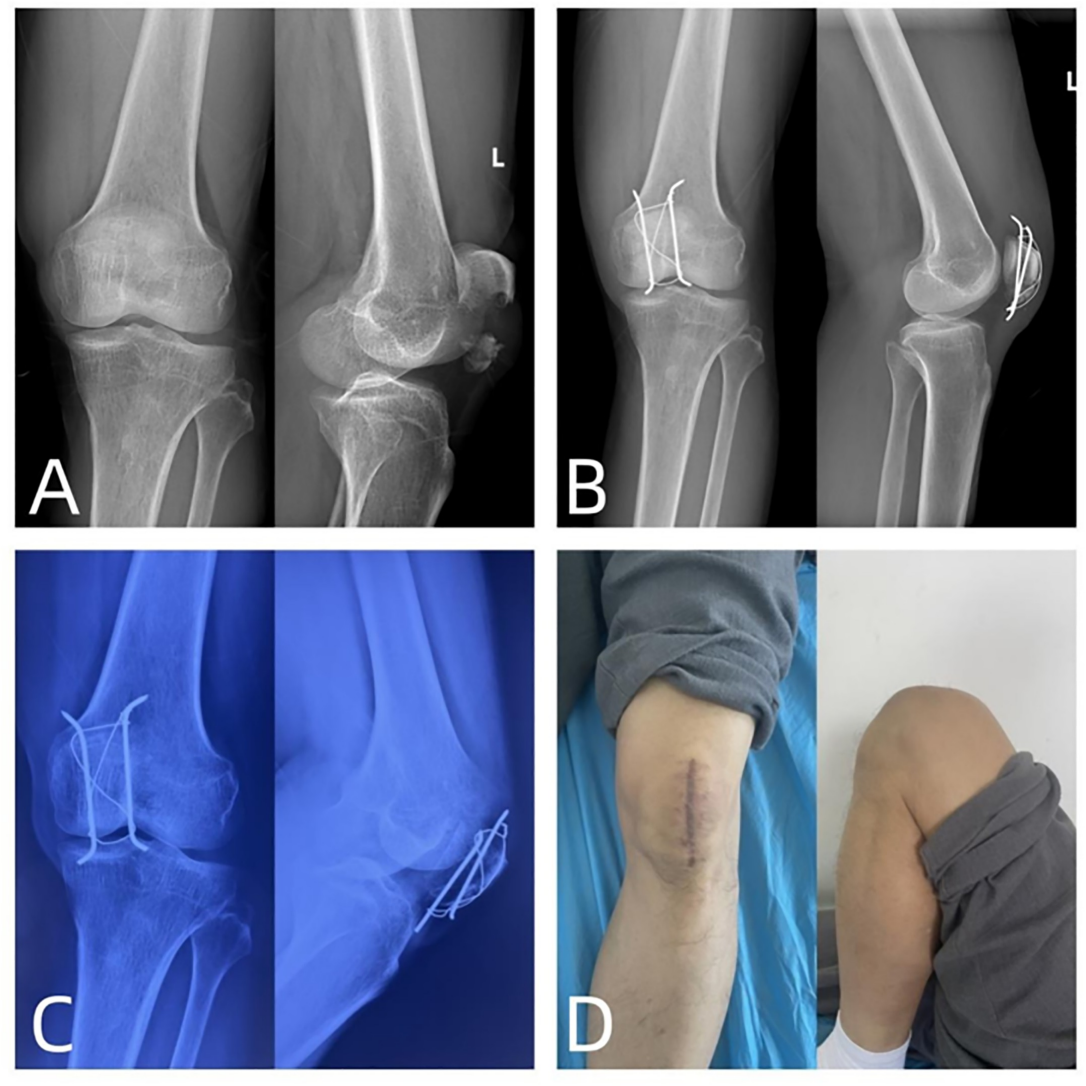
A patient is a 56-year-old male who sustained a comminuted fracture of the left patellar inferopolar region due to a fall. **A:** Preoperative left knee joint X-ray anteroposterior and lateral views. **B:** On the first day postoperative, anteroposterior and lateral X-ray images of the left knee joint were taken, and the anchor pin was not visualized on the X-ray. **C:** Eight months postoperative, anteroposterior and lateral X-rays of the left knee joint show that the fracture has clinically healed according to the standards. **D:** Functional status of the left knee joint mobility at eight months postoperatively.

Record the surgical duration, intraoperative blood loss, length of hospital stay, and surgical complications. The patients undergo regular outpatient X-ray examinations, compared to the X-rays taken before the injury (Fig 2A), to document fracture healing time (Fig 2B and 2C). During the last follow-up, we assessed the patients’ daily activity-related pain using the Knee Joint Visual Analog Scale (VAS). Knee joint function was evaluated using Knee Joint Range of Motion (ROM) (Fig 2D) and the Bostman score. The Bostman score ranges from 0 to 30 points, with a score of 28–30 indicating excellent, 20–27 indicating good, and less than 20 indicating poor outcomes. Postoperatively, anteroposterior and lateral X-rays of the knee joint were taken on the first day, at one month, and at two months after the surgery, and then weekly to evaluate fracture healing until union was achieved. The X-ray fracture healing criteria were based on a blurry or absent fracture line and the formation of continuous callus at the fracture site.

A typical case is shown in Fig 2.

## 3. Results

In this study, the surgical duration ranged from 30 to 46 minutes, with an average of 37.86±5.32 minutes. The intraoperative blood loss ranged from 20 to 45 mL, with an average of 33.29±8.15 mL. The length of hospital stay was 5.64±1.15 days. All 14 cases were successfully followed up for 12 to 18 months. All patients started to develop callus at the fracture ends at 4–6 weeks postoperatively, reaching clinical healing criteria at 10–14 weeks. Until the end of the follow-up period, no patients experienced loosening or detachment of the internal implants. The average time to fracture healing was 10.79±1.53 weeks. The mean postoperative range of motion was 131.07°±4.88°, and all followed-up patients were able to achieve full knee extension. The average Bostman score at the last follow-up was 28.29±0.83, with 12 cases (85.7%) rated as excellent and 2 cases (14.3%) rated as good according to the Bostman scale. The mean Visual Analog Scale (VAS) pain score was 0.36±0.63. Four patients reported mild anterior knee pain during daily activities. No cases of incision infection were observed, and the incisions in all 14 patients healed well postoperatively. X-ray examination at 3 months postoperatively showed that all patients met the clinical healing criteria. One patient experienced irritation from the internal fixation. Among the 14 patients, 6 chose to have the internal fixation removed after fracture healing. In one case, the decision was due to irritation from the internal fixation, while the others were based on socio-psychological factors (Table 1).

**Table 1:** Clinical results.

Number	Surgery Duration (minutes)	Blood Loss (milliliters)	Hospital Stay (days)	Fracture Healing Time (weeks)	Range of Motion	Bostman Score	VAS Score
1	40	35	7	12	0–130°	28	1
2	41	20	5	10	0–135°	28	0
3	30	45	4	13	0–130°	29	1
4	40	36	5	10	0–130°	28	0
5	37	45	4	9	0–135°	28	0
6	43	40	7	12	0–125°	27	1
7	35	37	6	11	0–130°	29	0
8	37	20	6	10	0–130°	28	0
9	28	25	5	8	0–140°	28	0
10	40	35	7	13	0–130°	29	0
11	43	30	6	11	0–135°	29	0
12	41	28	7	9	0–135°	28	0
13	30	40	6	12	0–130°	30	0
14	45	30	4	11	0–120°	27	2

## 4. Discussion

Inferior patellar pole fractures, occurring in the distal 1/4 of the patella, are a relatively rare type of fracture in clinical practice. Due to their anatomical and biomechanical characteristics, the fracture fragments are often comminuted and small. Additionally, they can experience displacement due to tension in the patellar tendon, classifying them as non-articular fractures [12]. The aim of the surgery is to restore the original extensor mechanism, enabling patients to engage in early functional rehabilitation exercises [13]. Therefore, treatment should focus on achieving early stable reduction of the fracture and restoring the continuity and strength of the extensor mechanism. Currently, although there are numerous surgical approaches for inferior patellar pole fractures, achieving anatomical reconstruction, effective fixation, and early postoperative rehabilitation remains a challenge [14,15].

Some experts have indicated that non-articular fractures of the inferior patellar pole can be excised, with resection and reconstruction of the tendon, followed by approximately 6 weeks of plaster external fixation. The excellent and good rate of joint scores is approximately 73.5% [16]. However, some have also argued that excision of the inferior patellar pole may result in patellar descent, disrupting the original anatomical relationships and altering the mechanical structure of the patellar joint surface. This could increase the load on the quadriceps femoris during knee joint movement and potentially lead to long-term adverse effects such as impaired knee extension function and traumatic patellofemoral arthritis [17,18]. Furthermore, postoperative healing after excision of the inferior patellar pole occurs through tendon-bone healing, a method that is often considered unreliable. A study by Veselko et al. [16] showed that patients who underwent preservation of the inferior patellar pole had significantly better knee joint function and fewer symptoms such as pain compared to those who underwent excision of the inferior patellar pole. Similar results were obtained in the study by Matejčić et al. [19]. In clinical practice, a greater number of individuals tend to favor preserving the inferior patellar pole and strive to restore the local original extensor mechanism and anatomical structure [20–22].

Song et al. [23] utilized the independent steel wire vertical technique combined with the Krachow suture technique to achieve effective fixation of comminuted fracture blocks, but wire breakage may occur. The tension band technique using Kirschner wires is the most commonly used and classic method for open reduction and internal fixation of patellar fractures. It can effectively stabilize patellar fractures by converting wire tension into axial pressure at the fracture site, thus increasing stability. However, it requires relatively intact bone quality, and when dealing with comminuted fractures of the inferior patellar pole, the Kirschner wires may loosen, making it difficult to achieve complete reduction and effective fixation [24,25]. Some articles have indicated that for comminuted fractures of the inferior patellar pole, the probability of requiring secondary surgical fixation after sole treatment with the tension band technique using Kirschner wires is approximately 21%-58% [26,27]. In our clinical practice, we have also observed that sole application of the tension band technique using Kirschner wires for fixing comminuted patellar fractures results in inadequate stability, particularly when the bone quality at the inferior patellar pole is poor. The Kirschner wires and wires used for internal fixation can easily cut through the porous bone, leading to loosening and failure of internal fixation. Gao et al. [22] proposed that fixation using the Krachow technique combined with the Nice knot achieved the expected results, but multiple operations on the patella may lead to iatrogenic patellar fractures. Yu et al. [2] pointed out that fixation of inferior patellar pole fractures using the two-anchor double-pulley technique poses risks of inadequate fracture fixation and fracture displacement. Therefore, in recent years, we have employed a fixation method combining anchor cross-stitch suturing and tension band wiring for comminuted fractures of the inferior patellar pole.

Suture anchors were initially used for repairing rotator cuff injuries, and with the advancement of medical technology, their application in surgical procedures has become increasingly widespread. The sutures of the anchors are composed of ultra-high molecular weight polyethylene fibers, offering advantages such as high strength and resistance to wear. This study employed the Kirschner wire tension band combined with anchor cross-stitch technique, which achieved favorable outcomes in clinical practice. This treatment method has the following advantages: (1) The Kirschner wire tension band, as the most classic treatment method for patellar fractures, is capable of converting tensile forces on the cortical bone surface into compression, promoting bone healing while retaining the patellar inferopolar fragments. (2) Inserting the anchor nail perpendicular to the transverse section of the proximal patellar fracture block can effectively ensure the stability of the anchor nail and does not cause irritation to the surrounding soft tissues.(3) Utilizing anchor screw cross-stitch fixation can encircle the shattered fragments of comminuted fractures of the inferior patellar pole, providing robust and effective internal fixation, capable of restoring knee extension, achieving anatomical realignment, and promoting bone-to-bone healing at the fracture site. Patients can engage in early postoperative functional rehabilitation exercises to prevent joint stiffness.(4) This treatment method is characterized by its simplicity of operation. The required materials are easily obtainable and available in most hospitals, without increasing surgical complexity.

The research findings of this study reveal that the Kirschner wire tension band combined with anchor cross-stitch technique not only restores the original extensor mechanism but also provides effective internal fixation for postoperative rehabilitation exercises. It demonstrates favorable clinical outcomes, low cost, allows for early postoperative rehabilitation exercises, and achieves satisfactory clinical results. In this study, all patients were able to commence passive quadriceps exercises, straight leg raises, and progressive knee flexion rehabilitation training after the first-day postoperative X-ray review. None of the patients experienced nonunion or loss of reduction fixation postoperatively, and there were no cases requiring secondary surgical intervention. However, a few patients self-reported mild anterior knee pain during squatting postoperatively, which may be associated with delayed initiation of rehabilitation exercises and irritation from the internal fixation. This study also has certain limitations. Firstly, due to time constraints, only patients with comminuted fractures of the inferior patellar pole were collected, resulting in a limited number of cases and no long-term follow-up. Secondly, comparisons with other treatment methods were not conducted.

## 5. Conclusion

In summary, the Kirschner wire tension band combined with anchor cross-stitch technique is a safe and effective treatment approach. It provides strong and reliable internal fixation for fractures of the patellar inferopolar region. This method is characterized by its simplicity in operation, dependable fixation, early postoperative initiation of functional rehabilitation exercises, and the potential for good recovery of knee joint function. It is a surgical approach worthy of clinical application.

## References

[1] Heusinkveld MHG, den Hamer A, Traa WA, Oomen PJA, Maffulli N. Treatment of transverse patellar fractures: a comparison between metallic and non-metallic implants. Br Med Bull. 2013;107:69–85. doi: 10.1093/bmb/ldt013 . 23620578

[2] Yu H, Dong H, Ruan B, Xu X, Wang Y, Hu L. Clinical Effect of Suture Anchor and Double-Pulley Technique in the Treatment of Inferior Patellar Fracture. Comput Math Methods Med. 2021;2021:4964195. doi: 10.1155/2021/4964195 . 35003320 PMC8741366

[3] Steinmetz S, Brügger A, Chauveau J, Chevalley F, Borens O, Thein E. Practical guidelines for the treatment of patellar fractures in adults. Swiss Med Wkly. 2020;150:w20165. doi: 10.4414/smw.2020.20165 . 31940427

[4] Cho J-W, Kim J, Cho W-T, Gujjar PH, Oh C-W, Oh J-K. Comminuted inferior pole fracture of patella can be successfully treated with rim-plate-augmented separate vertical wiring. Arch Orthop Trauma Surg. 2018;138(2):195–202. doi: 10.1007/s00402-017-2807-7 . 29058078

[5] Xie J, Fu Y, Li J, Yu H, Zhang Y, Jing J. Anchor and Krackow-"8" Suture for the Fixation of Distal Pole Fractures of the Patella: Comparison to Kirschner Wire. Orthop Surg. 2022;14(2):374–82. doi: 10.1111/os.13124 . 34964263 PMC8867415

[6] Gosal HS, Singh P, Field RE. Clinical experience of patellar fracture fixation using metal wire or non-absorbable polyester—a study of 37 cases. Injury. 2001;32(2):129–35. doi: 10.1016/s0020-1383(00)00170-4 . 11223044

[7] Kadar A, Sherman H, Drexler M, Katz E, Steinberg EL. Anchor suture fixation of distal pole fractures of patella: twenty seven cases and comparison to partial patellectomy. Int Orthop. 2016;40(1):149–54. doi: 10.1007/s00264-015-2776-9 . 25913264

[8] Oh H-K, Choo S-K, Kim J-W, Lee M. Internal fixation of displaced inferior pole of the patella fractures using vertical wiring augmented with Krachow suturing. Injury. 2015;46(12):2512–5. doi: 10.1016/j.injury.2015.09.026 . 26482481

[9] Matejčić A, Ivica M, Jurišić D, Ćuti T, Bakota B, Vidović D. Internal fixation of patellar apex fractures with the basket plate: 25 years of experience. Injury. 2015;46 Suppl 6:S87–S90. doi: 10.1016/j.injury.2015.10.068 . 26584729

[10] Anand A, Kumar M, Kodikal G. Role of suture anchors in management of fractures of inferior pole of patella. Indian J Orthop. 2010;44(3):333–5. doi: 10.4103/0019-5413.65149 . 20697489 PMC2911936

[11] He Q-F, Pan G-B, Yu Z-F, Yao W-X, Zhu L-L, Luo C-F, et al. Novel Rim Plating Technique for Treatment of the Inferior Pole Fracture of the Patella. Orthop Surg. 2021;13(2):651–8. doi: 10.1111/os.12876 . 33619908 PMC7957411

[12] Bui CN, Learned JR, Scolaro JA. Treatment of Patellar Fractures and Injuries to the Extensor Mechanism of the Knee: A Critical Analysis Review. JBJS Rev. 2018;6(10):e1. doi: 10.2106/JBJS.RVW.17.00172 . 30277900

[13] Wright PB, Kosmopoulos V, Coté RE, Tayag TJ, Nana AD. FiberWire is superior in strength to stainless steel wire for tension band fixation of transverse patellar fractures. Injury. 2009;40(11):1200–3. doi: 10.1016/j.injury.2009.04.011 . 19524229

[14] Kim K-S, Suh D-W, Park S-E, Ji J-H, Han Y-H, Kim J-H. Suture anchor fixation of comminuted inferior pole patella fracture-novel technique: suture bridge anchor fixation technique. Arch Orthop Trauma Surg. 2021;141(11):1889–97. doi: 10.1007/s00402-020-03671-5 . 33125547

[15] Zhu W, Xie K, Li X, Li L, Yang J, Xu L, et al. Combination of a miniplate with tension band wiring for inferior patellar pole avulsion fractures. Injury. 2020;51(3):764–8. doi: 10.1016/j.injury.2020.01.028 . 32005322

[16] Veselko M, Kastelec M. Inferior patellar pole avulsion fractures: osteosynthesis compared with pole resection. Surgical technique. J Bone Joint Surg Am. 2005;87 Suppl 1(Pt 1):113–21. doi: 10.2106/JBJS.D.02631 . 15743853

[17] Egol K, Howard D, Monroy A, Crespo A, Tejwani N, Davidovitch R. Patella fracture fixation with suture and wire: you reap what you sew. Iowa Orthop J. 2014;34:63–7. . 25328461 PMC4127725

[18] Nathan ST, Fisher BE, Roberts CS, Giannoudis PV. The management of nonunion and delayed union of patella fractures: a systematic review of the literature. Int Orthop. 2011;35(6):791–5. doi: 10.1007/s00264-010-1105-6 . 20680273 PMC3103972

[19] Matejcić A, Puljiz Z, Elabjer E, Bekavac-Beslin M, Ledinsky M. Multifragment fracture of the patellar apex: basket plate osteosynthesis compared with partial patellectomy. Arch Orthop Trauma Surg. 2008;128(4):403–8. doi: 10.1007/s00402-008-0572-3 . 18270723

[20] Camarda L, La Gattuta A, Butera M, Siragusa F, D’Arienzo M. FiberWire tension band for patellar fractures. J Orthop Traumatol. 2016;17(1):75–80. doi: 10.1007/s10195-015-0359-6 . 26142873 PMC4805633

[21] O’Donnell R, Lemme NJ, Marcaccio S, Walsh DF, Shah KN, Owens BD, et al. Suture Anchor Versus Transosseous Tunnel Repair for Inferior Pole Patellar Fractures Treated With Partial Patellectomy and Tendon Advancement: A Biomechanical Study. Orthop J Sports Med. 2021;9(8):23259671211022245. doi: 10.1177/23259671211022245 . 34423057 PMC8371734

[22] Gao Z, Long N, Yao K, Cai P, Dai Y, Yu W, et al. A Novel Technique for the Treatment of Inferior Pole Fractures of the Patella: A Preliminary Report. Orthop Surg. 2022;14(11):3092–9. doi: 10.1111/os.13518 . 36196019 PMC9627058

[23] Song HK, Yoo JH, Byun YS, Yang KH. Separate vertical wiring for the fixation of comminuted fractures of the inferior pole of the patella. Yonsei Medical Journal. 2014;55(3):785–91. doi: 10.3349/ymj.2014.55.3.785 . 24719149 PMC3990064

[24] Lin D-F, Yang W-Q, Zhang P-P, Lv Q, Jin O, Gu J-R. Clinical and prognostic characteristics of 158 cases of relapsing polychondritis in China and review of the literature. Rheumatol Int. 2016;36(7):1003–9. doi: 10.1007/s00296-016-3449-8 . 26951051

[25] Wild M, Fischer K, Hilsenbeck F, Hakimi M, Betsch M. Treating patella fractures with a fixed-angle patella plate-A prospective observational study. Injury. 2016;47(8):1737–43. doi: 10.1016/j.injury.2016.06.018 . 27354301

[26] Wurm S, Bühren V, Augat P. Treating patella fractures with a locking patella plate—first clinical results. Injury. 2018;49 Suppl 1:S51–S5. doi: 10.1016/S0020-1383(18)30304-8 . 29929694

[27] Ling M, Zhan S, Jiang D, Hu H, Zhang C. Where should Kirschner wires be placed when fixing patella fracture with modified tension-band wiring? A finite element analysis. J Orthop Surg Res. 2019;14(1):14. doi: 10.1186/s13018-019-1060-x . 30634995 PMC6329102

